# Nuclear Magnetic Resonance Treatment Induces ßNGF Release from Schwann Cells and Enhances the Neurite Growth of Dorsal Root Ganglion Neurons In Vitro

**DOI:** 10.3390/cells13181544

**Published:** 2024-09-13

**Authors:** Anda Rad, Lukas Weigl, Bibiane Steinecker-Frohnwieser, Sarah Stadlmayr, Flavia Millesi, Maximilian Haertinger, Anton Borger, Paul Supper, Lorenz Semmler, Sonja Wolf, Aida Naghilou, Tamara Weiss, Hans G. Kress, Christine Radtke

**Affiliations:** 1 Research Laboratory of the Department of Plastic, Reconstructive and Aesthetic Surgery, Medical University of Vienna, Waehringerstrasse 18-20, 1090 Vienna, Austria; anda.rad@meduniwien.ac.at (A.R.); sarah.stedlmayr@meduniwien.ac.at (S.S.); flavia.millesi@meduniwien.ac.at (F.M.); maximilian.haertinger@meduniwien.ac.at (M.H.); anton.bogner@meduniwien.ac.at (A.B.); paul.supper@meduniwien.ac.at (P.S.); lorenz.semmler@meduniwien.ac.at (L.S.); sonja.wolf@meduniwien.ac.at (S.W.); a.naghilouye.hidaji@lacdr.leidenuniv.nl (A.N.); christine.radtke@meduniwien.ac.at (C.R.); 2 Clinical Department of Special Anesthesia and Pain Therapy, Department of Anaesthesia, Intensive Care Medicine and Pain Medicine, Medical University of Vienna, Waehringerstrasse 18-20, 1090 Vienna, Austria; hans.georg.kress@chello.at; 3 Ludwig Boltzmann Institute for Arthritis and Rehabilitation, Hofmanning 214, 8962 Groebming, Austria; 4 Medical Systems Biophysics and Bioengineering, Leiden Academic Centre for Drug Research, Leiden University, 2333 CC Leiden, The Netherlands

**Keywords:** peripheral nerve regeneration, low nuclear magnetic resonance therapy, Schwann cell, DRG neuron, cytokines, neurite outgrowth, nociception, TRPV1

## Abstract

Peripheral nerve regeneration depends on close interaction between neurons and Schwann cells (SCs). After nerve injury, SCs produce growth factors and cytokines that are crucial for axon re-growth. Previous studies revealed the supernatant of SCs exposed to nuclear magnetic resonance therapy (NMRT) treatment to increase survival and neurite formation of rat dorsal root ganglion (DRG) neurons in vitro. The aim of this study was to identify factors involved in transferring the observed NMRT-induced effects to SCs and consequently to DRG neurons. Conditioned media of NMRT-treated (CM NMRT) and untreated SCs (CM CTRL) were tested by beta-nerve growth factor (ßNGF) ELISA and multiplex cytokine panels to profile secreted factors. The expression of nociceptive transient receptor potential vanilloid 1 (TRPV1) channels was assessed and the intracellular calcium response in DRG neurons to high-potassium solution, capsaicin or adenosine triphosphate was measured mimicking noxious stimuli. NMRT induced the secretion of ßNGF and pro-regenerative-signaling factors. Blocking antibody experiments confirmed ßNGF as the main factor responsible for neurotrophic/neuritogenic effects of CM NMRT. The TRPV1 expression or sensitivity to specific stimuli was not altered, whereas the viability of cultured DRG neurons was increased. Positive effects of CM NMRT supernatant on DRG neurons are primarily mediated by increased ßNGF levels.

## 1. Introduction

Peripheral nerve injuries (PNIs) continue to be a major clinical challenge. Despite advancements in microsurgical techniques to improve nerve repair, patients hardly regain full motor and sensory functions [1]. Therefore, innovative treatment strategies are needed to accelerate nerve regeneration and improve functional recovery after injury.

Damage to peripheral nerves triggers a complex cellular and molecular response distal to the injury site [2,3]. The important role of Schwann cells (SCs) in nerve regeneration has been extensively investigated, demonstrating their plasticity and ability to switch into a repair-supportive cellular state post injury [4]. Repair SCs upregulate cytokines and chemokines that stimulate the invasion of immune cells to the site of injury [5,6]. Recent studies suggest that repair SCs also produce factors involved in immunomodulatory functions that can shape the inflammatory environment towards regeneration [7,8,9]. Furthermore, repair SCs provide important support for regenerating nerves such as the formation of cords of cells (Bands of Büngner) to guide the regrowing axons towards their target organ, and the secretion of a variety of neurotrophic factors [2,10,11]. The beta-nerve growth factor (ßNGF) is one of the first and best-characterized neurotrophic factors with essential functions during neuronal development, maintenance and regeneration [12,13]. After nerve injury, the release of ßNGF from SCs is important to support axon sprouting and guidance and eventually improve functional recovery after injury [14,15,16].

However, neurotrophic factors can also induce peripheral neuropathic pain [17,18,19]. Pain usually starts with an activation of nociceptors, a subset of dorsal root ganglion (DRG) neurons that respond to different noxious stimuli during tissue damage and inflammation [20]. Important receptor molecules for nociceptors are acid-sensing ion channels, P2X3 receptors that are activated by adenosine triphosphate (ATP), and transient receptor potential vanilloid 1 (TRPV1) channels sensitive to high temperature and capsaicin. Thus, treatment approaches targeting axon regeneration should not increase the nociceptor sensitivity to noxious stimuli which then could contribute to the development of peripheral neuropathic pain.

Previously, low-intensity nuclear magnetic resonance therapy (NMRT) has been shown to improve the condition of patients suffering from musculoskeletal disorders and severe back pain [21]. On a cellular level, NMRT was able to enhance the proliferation of cultured chondrocytes and osteoblast cells in vitro [22]. Furthermore, it was able to modulate the expression of growth factors and miRNAs in chondrocytes [23,24], change the expression of proteins involved in cell adhesion and movement of skin fibroblasts [25], and modulate the expression and oscillation of specific core clock genes and Hif isoforms in zebrafish-derived fibroblasts [26]. Furthermore, our recently published study on the effect of NMRT on rat SCs and DRG neuron cell cultures revealed that the supernatant of NMRT-treated SCs enhanced the survival and neurite formation of DRG neurons [27]. Interestingly, it was suggested that the stimulation by a low-intensity magnetic field could promote the release of NGF in a mouse model through the regulation of growth factors such as TGFß [28,29,30]. These findings support our hypothesis that NMRT can induce the production of pro-regenerative factors in SCs and could offer a non-invasive treatment option to support the regeneration process of injured nerves by SC stimulation. To assess a future in vivo application of NMRT, we here also analyzed the levels of growth factors and cytokines released by NMRT-treated SCs and investigated whether NMRT-exposed SC supernatant might modulate TRPV1 which is involved in nociception on cultured DRG neurons.

## 2. Materials and Methods

### 2.1. Animals

Female and male Lewis Wistar rats (20–25 weeks old) were sacrificed by guillotine decapitation under deep isoflurane anesthesia, and sciatic nerves and DRG were harvested. Breeding, housing, transport and sacrifice of animals were conducted in compliance with Austrian Animal Testing Law (TVG 2012, §2, 1.c) and Article 3 of the Directive 2010/63/EU of the European Parliament and of the Council on the Protection of Animals Used for Scientific Purposes (European Parliament, 2010).

### 2.2. Isolation and Culture of Rat Schwann Cells

The isolation, culture and purification of SCs were performed as previously described [27,31,32]. Sciatic nerves were excised and washed in 1× Dulbecco’s phosphate buffer saline (1× PBS, GIBCO, Waltham, MA, USA) augmented with+ 1% penicillin-streptomycin (P/S, GIBCO). The fascicles were pulled out of the epineurium and were digested overnight in MEMα (GIBCO) supplemented with 10% fetal calf serum (FCS, LINARIS, Wertheim, Germany), 1% penicillin, 1% sodium pyruvate solution (GIBCO), 2.5% 4-(2-hydroxyethyl)-1-piperazineethanesulfonic acid buffer solution (HEPES, SIGMA-Aldrich, Burlington, MA, USA), 0.125% (*w*/*v*) collagenase type IV (GIBCO), 1.25 U/mL dispase II (SIGMA-Aldrich) and 3 mM calcium chloride (Merck, Kenilworth, NJ, USA) at 37 °C and 5% CO_2_. The cell suspension derived from nerve digest was seeded on 0.01% poly-L-lysine hydrobromide (PLL) (SIGMA-Aldrich) and 5 µg/mL laminin (SIGMA-Aldrich) coated dishes in SC culture medium (SCEM) consisting of MEMα supplemented with 1% P/S, 1% sodium pyruvate solution, 2.5% HEPES, 0.5% N-2 Supplement (GIBCO), 2 μM forskolin (SIGMA-Aldrich), 10 ng/mL recombinant heregulinβ-1 (PeproTech, Rocky Hill, NJ, USA), 10 ng/mL recombinant fibroblast growth factor basic (FGF-basic) (PeproTech), 5 ng/mL platelet derived growth factor-AA (PDGF- AA) (PeproTech) and 5% FCS. The medium was changed three times a week. For the separation of SCs from rat fibroblasts (FBs), a two-step enrichment procedure according to Weiss et al. [33] was used. We took advantage of the differential adhesion properties of SCs and FBs resulting in a culture purity of about 95%. SCs were passaged upon reaching 80–90% confluence and SC cultures in passage 3 (p3) were used for experimentation. Phase contrast images of SCs were regularly taken with a benchtop microscope (NIKON Eclipse Ts25, Tokyo, Japan).

### 2.3. Nuclear Magnetic Resonance Treatment Regimen

**Figure 1:**
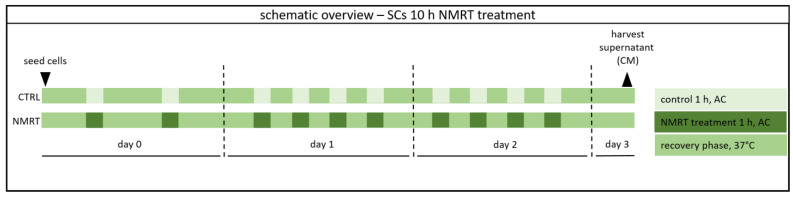
Schematic timetable of the NMRT treatment. SCs were subjected to 10 NMRT treatment cycles: two cycles on day 0, four cycles on day 1 and four cycles on day 2; one treatment cycle consisted of 1 h NMRT at ambient conditions (AC: outside the incubator at room temperature) followed by at least a 1.5 h recovery phase in the incubator at 37 °C and 5% CO_2_; control cultures were also maintained at ambient conditions during the respective NMRT periods; after the last treatment cycle, cells were incubated at 37 °C and 5% CO_2_ overnight before further analysis.

Highly enriched SC cultures were treated with a therapeutic low-intensity NMRT device adapted for cell cultures (MBST, MedTec Medizintechnik GmbH, Wetzlar, Germany). The device parameters were a field of 0.4 mT and a radiofrequency of 16 kHz [29]. One treatment cycle consisted of 1 h NMRT at ambient conditions followed by a 1.5 h recovery phase in the incubator at 37 °C and 5% CO_2_. SCs were subjected to 10 NMRT treatment cycles as shown in Figure 1. In parallel, control cultures were also maintained at ambient conditions during the respective NMRT periods. After the last treatment cycle, cells were incubated at 37 °C and 5% CO_2_ overnight before further analysis.

### 2.4. Harvest of Schwann-Cell-Conditioned Medium

Conditioned medium of primary SC cultures after a 10 h NMRT treatment (CM NMRT) and of untreated control SC cultures (CM CTRL) were harvested and centrifuged at 3000× *g* for 10 min at 4 °C to remove dead cells and debris. The supernatant was aliquoted and kept at −80 °C until further use. If the experiment required it, the ßNGF-neutralizing antibody was added to the conditioned medium in a concentration of 50 ng/mL. This specific antibody was purchased by SIGMA-Aldrich.

### 2.5. ßNGF ELISA and Cytokine Profiling of Conditioned Schwann Cell Media

To determine the levels of ßNGF in the SC-conditioned-medium, a rat ßNGF ELISA kit (Thermo Fischer, Waltham, MA, USA) was utilized. The frozen supernatant samples, CM CTRL and CM NMRT, were thawed slowly at 4 °C and their ßNGF level was determined according to the manufacturer’s instructions. Substrate absorbance was recorded at 450 nm on an EnSpire microplate reader (Perkin Elmer, Waltham, MA, USA). All samples were assayed in duplicate.

To determine the expression levels of multiple cytokines in SC-conditioned-medium, we used the proteome profiler rat XL cytokine array (R&D Systems, Wiesbaden, Germany), which simultaneously detects the level of 79 rat cytokines. CM CTRL and CM NMRT supernatant samples were thawed slowly at 4 °C and the determination of cytokines was conducted according to the manufacturer’s manual. In addition, SCEM was used as a further control. The membranes were visualized with the Fusion-Pulse imaging system (Vilber Lourmat, Marne-la-Vallée, France). Samples from three individual donors were assayed in duplicate. Analysis was performed with Vilber’s Bio-1D analysis software (v.15.07).

### 2.6. Isolation and Culture of Dorsal Root Ganglion Neurons

Thoracic and lumbar DRG pairs were harvested from the spinal roots and washed in 1× PBS with+ 1% P/S. The DRGs were digested first in a Ca^2+^ free Tyrode solution (150 mM NaCl, 4 mM KCl, 2 mM MgCl_2_, 10 mM glucose, 10 mM HEPES, pH = 7.4) containing collagenase IA (770 U/mL, SIGMA-Aldrich) and dispase II (1.5 U/mL, Roche, Basel, Switzerland) at 37 °C for 45 min and then for 20 min in trypsin (745U/mL, from bovine pancreas, Worthington, OH, USA). After trituration, the cell suspension was washed twice in Ca^2+^-free Tyrode solution and freshly dissociated DRG neurons were seeded in a 1:1 mix of CM CTRL/DRG medium (DRGM) or in a CM 1:1 mix of CM NMRT/DRGM on 8 wells chamber slides (ibidi, Gräfelfing, Germany), glass plates or petri dishes. DRGM consisted of NeurobasalTM-A medium (GIBCO) supplemented with 1× B27 supplement (Invitrogen, Waltham, MA, USA) and 2 mM L-glutamine (Invitrogen). DRG neurons were cultured at 37 °C and 5% CO_2_ for 24–30 h before the analysis. DRG cultures were visually assessed regularly via a benchtop microscope (NIKON Eclipse Ts2R).

### 2.7. Immunofluorescence Staining for Schwann Cell Phenotyping and DRG Neuron Feature Analysis

All procedures were carried out at room temperature unless otherwise noted. All antibody details are listed in Supplementary Table S1. For immunofluorescence staining, first SCs or DRG neuron cultures were washed with 1× PBS and fixed with 4.5% formaldehyde solution (SAV Liquid Production GmbH, Flintsbach a. Inn, Germany) for 15 min. The washing step involved a sequential incubation with 1× PBS for 5 min each. Blocking and permeabilization was performed in one step with 1× PBS containing 1% bovine serum albumin (BSA, SIGMA-Aldrich), 0.3% Triton X-100 (SIGMA-Aldrich) and 5% goat serum (DAKO, Glostrup, Denmark) for 20 min. Subsequently, the cells were incubated with primary antibodies in 1× PBS containing 1% BSA, 0.1% Triton X-100 and 1% goat serum overnight at 4 °C. Immunostaining was performed using SC markers S100 protein (Dako, Auckland, New Zealand), the glial cell-associated transcription factor SOX10 (Santa Cruz Biotechnology, Dallas, TX, USA) and the intermediate filament vimentin (VIME) (ThermoFisher) to determine the purity of the SC cultures.

Cells were washed and incubated with the respective secondary antibodies for 1 h. For nuclear staining, 50 µg/mL 4,6-diamidino-2-phenylindole solution (DAPI, Thermo Fisher) was added for 10 min. After washing, the cells were embedded in fluoromount-G mounting medium (Invitrogen). Immunofluorescence images were taken using an Eclipse Ti (Nikon, Tokyo, Japan) microscope. For SCs, phenotyping cells from three different donors were analyzed. For DRG neuron features, analysis cells from four different donors were tested. Images are depicted as maximum intensity projections of total z-stacks; contrast and brightness were adapted in a homogenous manner; and pseudo-coloring was applied to make multicolor figures comprehensible for colorblind readers.

### 2.8. Intracellular Calcium Imaging in Dorsal Root Ganglion Neurons

For determination of intracellular calcium concentration ([Ca^2+^]_i_) as a response to either high-potassium solution (HK: Tyrode solution with 90 mM NaCl and 60 mM KCl), 3 µM capsaicin (Cap, SIGMA-Aldrich) or 10 µM ATP (SIGMA-Aldrich), DRG neurons were incubated 35 to 45 min in loading buffer (Tyrode’s solution with 3.5 µM Fura2/AM; Molecular Probes, Waltham, MA, USA) and 0.025% Pluronic at room temperature. Coverslips were washed with Tyrode solution and placed into a perfusion chamber of a fluorescence microscope at 40× magnification. HK, ATP and Cap were applied directly to a cell with a 7-channel superfusion pipette, and fluorescence images were taken at excitation wavelengths of 340 and 380 nm with an emission wavelength of 510 nm. Images were recorded with a sample interval of 1 to 10 s and analyzed with the Visiview 3.1.0software (Visitron Systems, Puchheim, Germany). Calibration of fluorescence signals to calculate [Ca^2+^]_i_ was performed according to Grynkiewicz et al. and Thomas and Delaville [34,35,36,37]. Resting [Ca^2+^]_i_ was defined by the average of the first 10 data points before the application of any treatment substances. The value of a [Ca^2+^]_i_ transient was determined by the peak value reached within the time of substance application. After complete washout of the substance and return of [Ca^2+^]_i_ to baseline values, the next substance or trigger was applied. From each individual (*n* = 4) and of each conditioned medium treatment, at least five cells were tested.

### 2.9. Patch Clamp Experiments for Current Measurement in Dorsal Root Ganglion Neurons

Whole-cell patch clamp experiments were performed using a HEKA EPC-9 patch clamp amplifier with the patchmaster V2.90 software package (HEKA Elektronik, Lambrecht, Germany). Patch pipettes were pulled from non-heparinized micro hematocrit glass capillaries (Hecht, Pfaffenhofen, Germany) to a resistance of 1.0–2.0 MOhm. Current measurement was performed in a bath solution containing 150 mM NaCl, 5 mM KCl, 1 mM MgCl_2_, 2 mM CaCl_2_, 10 mM HEPES, 10 mM glucose, pH 7,3; pipette solution contained: 125 mM KCl, 10 mM EGTA/2Na^+^, 2 mM MgCl_2_, 3 mM CaCl_2_, 5 mM ATP/Na^+^, 0.2 mM GTP/Na^+^ and 10 mM HEPES, pH 7.2. The resting membrane potential was assessed in the current clamp mode (I = 0) immediately after rupture of the patch. The capacitance of the cells was measured using a sine wave protocol and the lock-in function of the patchmaster software. The diameter of cells was determined with a reticule built into the eyepiece of the microscope. For oval-shaped cells, the mean value of the short and long axis was used.

### 2.10. Reverse Transcription Polymerase Chain Reaction

RNA isolation of DRG neurons cultured in CM CTRL and CM NMRT was performed with the Arcturus™ PicoPure™ RNA Isolation Kit (Thermo Fischer) with digestion of genomic DNA by DNase I (Qiagen, Venlo, The Netherlands) according to the manufacturer’s manual. Subsequently, RNA concentration was measured photometrically (Nanophotometer, Implen, Munich, Germany) and 300 ng RNA was reverse transcribed (iScriptTM cDNA Synthesis Kit, BioRad, Hercules, CA, USA). The RT-qPCR was performed using SYBR Green Supermix (BioRad) and the 7500 Fast Real-Time PCR System (Applied Biosystems, Waltham, MA, USA). The respective primer sequences are listed in Supplementary Table S2. Each analysis was performed in duplicate. The quantities of target gene expression (CT-values) were calculated relative to the geometric mean of the internal control genes, GAPDH and β-actin, and presented as gene expression fold change between NMRT-treated and control samples (2^−(ΔΔCT)^).

### 2.11. Western Blot

DRG neurons cultured for 24 h in CM CTRL and CM NMRT were lysed with neuronal protein extraction reagent (N-PER buffer, Thermo Fischer) according to the manufacturer’s manual. The proteins were mixed with SDS-loading buffer (Laemli buffer, BioRad), denatured for 5 min at 95 °C, followed by separation on a 4–20% SDS-PAGE (BioRad) and blotted onto PVDF membranes (BioRad). Membranes were blocked using 1× tris buffered saline with 0.1% Tween 20 (TBS-T) with 5% BSA for 1 h and incubated with primary antibodies (TRPV1, alomone labs, Jerusalem, Israel, 1:500) in 1× TBS-T with 5% BSA overnight at 4 °C. After washing, respective secondary antibodies were added for 1 h at room temperature. For normalization, GAPDH (Cell Signaling, Waltham, MA, USA, 1:1000) was used as a reference protein. The respective antibodies and dilutions are listed in Supplementary Table S1. The blots were analyzed with the Odyssey CL × Imaging System and software V5.2 (Li–Cor Biosciences, Lincoln, NE, USA). Analysis was performed with the Image J software vs. 1.53 and the mean grey value of each band was measured.

### 2.12. Statistical Analysis

The significance of differences of the measured parameters between experimental conditions were evaluated with GraphPad Prism Version 9.4.0 (GraphPad Software, Boston, MA, USA). The normal distribution of the data was assessed by quantile–quantile plots; after that a ROUT outlier test (Q = 1) was performed. The significance of differences of the measured parameters (e.g., ßNGF and cytokine profiler) between experimental conditions (CM CTRL and CM NMRT) was evaluated using a paired *t*-test approach. A one-way ANOVA succeeded by Tukey’s all-pairs comparisons was applied for the blocking antibody experiments and TRPV1 experiments. For all experiments, an additional non-parametric Kruskal–Wallis test and Dunn’s multiple comparison tests were executed to assess the sensitivity and robustness of the data. If the data were not distributed normally, the statistical analysis after the outliner test was carried out by means of the Kruskal–Wallis test and Dunn’s post hoc test. The results are depicted as mean values ± standard deviation (SD) or median + 95 % CI; * *p* < 0.05, ** *p* < 0.01, *** *p* < 0.001.

## 3. Results

### 3.1. Characterization of Schwann Cells Used for the NMRT Treatment and Conditioned Media Harvest

**Figure 2:**
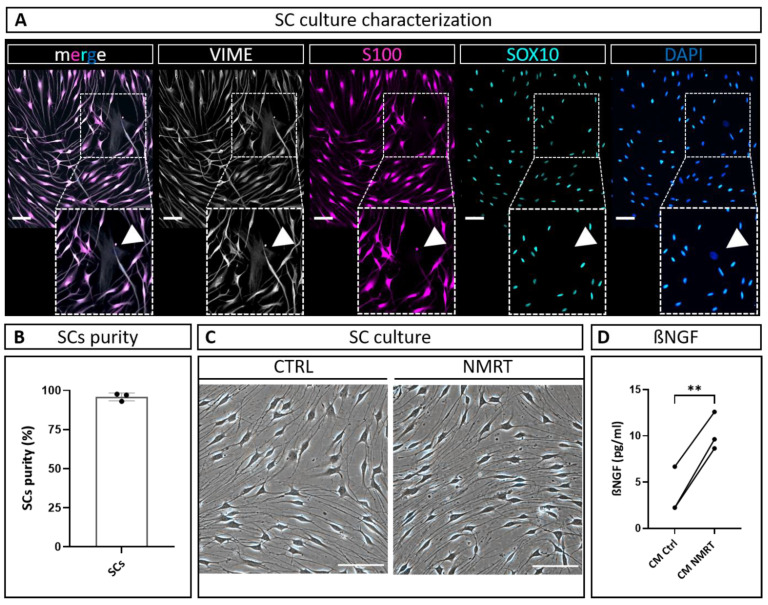
Characterization of SC cultures and analysis of ßNFG concentration in response to NMRT. (**A**) Representative fluorescence images of the primary SC culture used for NMRT treatment and conditioned media harvest stained for intermediate filament vimentin (VIME, white), S100 (magenta), SOX10 (green) and DAPI (blue); filled arrowheads indicate a SOX10-/S100-/VIME+/DAPI+ fibroblast. Scale bars 50 µm. (**B**) SC culture purity was calculated as percentage of SOX10+/DAPI+ cells (SCs) from DAPI+ cells (all cells). (**C**) Representative phase contrast images illustrating the similar morphology of control SC cultures and NMRT-treated SC cultures after a 10 h treatment cycle. Scale bars 50 µm. (**D**) ßNGF concentration measured in CM CTRL and CM NMRT; values that correspond to the same donor are connected by a line; data are depicted as average values of measured duplicates for each donor (*n* = 3); paired *t*-test; ** *p* < 0.01.

In order to verify the quality of three independent primary rat SC cultures used for NMRT treatment and conditioned media harvest, we characterized the expression of typical SC markers and determined the SC culture purity. The immunostaining analysis for SC markers S100 protein and the glial cell-associated transcription factor SOX10 demonstrated that the cultures were composed of highly enriched SC populations (Figure 2A). The few fibroblasts present in SC cultures were negative for S100 and SOX10, while both SCs and fibroblasts express the intermediate filament vimentin (VIME) (Figure 2A). To determine the purity of SC cultures, we quantified the number of SCs (SOX10 positive cells) from all cells (DAPI positive cells), demonstrating a purity of 95.8% ± 2.5% (Figure 2B). Phase contrast images of NMRT-treated and untreated SC cultures showed that SCs possessed the characteristic spindle-shaped morphology with di- and tri-polar extensions and formed the typical swirled parallel alignment in both conditions (Figure 2C). These findings confirm the identity and high purity of the SC culture used for the NMRT treatment and subsequent harvest of conditioned media for downstream experiments. No morphological changes by NMRT treatment have been detected.

### 3.2. Nuclear Magnetic Resonance Treatment Enhances the Secretion of ßNGF in Schwann Cells

Since ßNGF plays such an important role in nerve regeneration and was suggested to be released by a low-intensity magnetic field [30], we analyzed the levels of ßNGF in the conditioned medium of untreated SCs (CM CTRL) and NMRT-treated SCs (CM NMRT). The results revealed a significantly higher concentration of ßNGF in CM NMRT compared with CM CTRL (Figure 2D).

### 3.3. The Concentration of ßNGF Determined in CM NMRT Was Sufficient to Increase the Neurite Outgrowth of Dorsal Root Ganglion Neurons

**Figure 3:**
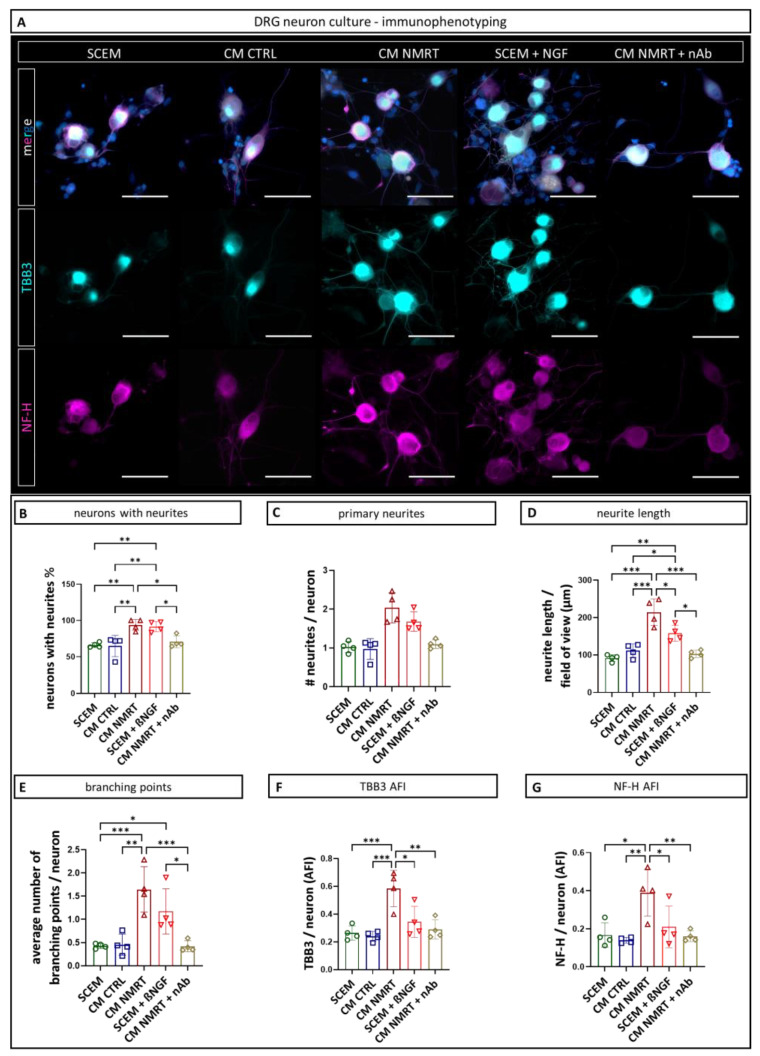
Comparison of DRG neuron cultures and neurite features in response to conditioned media and ßNGF presence/elimination. (**A**) Representative immunofluorescence images of DRG neurons cultured in SCEM, CM CTRL, CM NMRT, SCEM + 10 pg/mL ßNGF and CM NMRT + 50 ng/mL ßNGF neutralizing antibody (nAb) after the 24 h of culture stained for early neuronal marker tubulin β-3 (TBB3, cyan), axonal maturation marker neurofilament-heavy polypeptide (NF-H, magenta) and DAPI (blue). An enhanced neurite outgrowth of DRG neurons was observed when cultured in CM NMRT and SCEM + ßNGF, but not when DRG neurons were cultured in CM NMRT + nAb. Scale bars represent 50 µm. (**B**) Percentage of DRG neurons with neurites per donor; data are depicted as mean ± SD (*n* = 4). (**C**) Quantification of the mean number of primary neurites per DRG neuron. (**D**) The mean length of all neurites per DRG neuron (field of view). (**E**) Number of neurite branching points per DRG neuron. (**F**) Quantification of the TBB3 mean fluorescence intensity (given in average fluorescence intensity (AFI)) per DRG neuron. (**G**) Quantification of the NF-H mean fluorescence intensity per DRG neuron; data are depicted as mean values for each donor ± SD (*n* = 4), one-way ANOVA, * *p* < 0.05 ** *p* < 0.01, *** *p* < 0.001.

Next, we investigated whether the determined concentration of ßNGF in CM NMRT solely was sufficient to reproduce the beneficial effect of CM NMRT treatment on DRG neurons. Therefore, we cultured freshly isolated DRG neurons in pure SCEM, CM CTRL, CM NMRT and pure SCEM + 10 pg/mL ßNGF for 24 h, respectively. As an additional control, a ßNGF-neutralizing antibody (nAb, 50 ng/mL) was mixed with CM NMRT (CM NMRT + nAb) before it was added to the DRG neurons. For qualitative assessment of neurite outgrowth, we stained the DRG neuron cultures for the axonal protein tubulin ß-3 (TBB3), typically expressed in developing axons, and for neurofilament-heavy polypeptide (NF-H), a marker for maturing neurons (Figure 3A). The immunofluorescence images outline that the DRG neurite formation was strongest in the CM NMRT and SCEM + ßNGF condition, and that the neutralizing antibody counteracts this effect (Figure 3A). To this end, we quantified the number of neurons with neurites, the number of primary neurites, the mean number of branching points per DRG neuron and the mean neurite length per field of view (Figure 3B–E). Furthermore, the mean fluorescence intensities of TBB3 and NF-H were analyzed (Figure 3F,G). All tested parameters were significantly increased in CM NMRT and SCEM + ßNGF and reduced to control levels, when the neutralizing antibody was added (Figure 3B). Our results corroborate the importance of ßNGF for DRG neuron regeneration, as has been demonstrated in previous studies [38]. These findings suggest that the NMRT-induced increase in ßNGF secretion by SCs mediates the beneficial effect on DRG neuron neurite outgrowth. All data can be found in Supplementary Table S3.

### 3.4. Nuclear Magnetic Resonance Treatment Enhances the Release of Cytokines Involved in Nerve Regeneration

**Figure 4:**
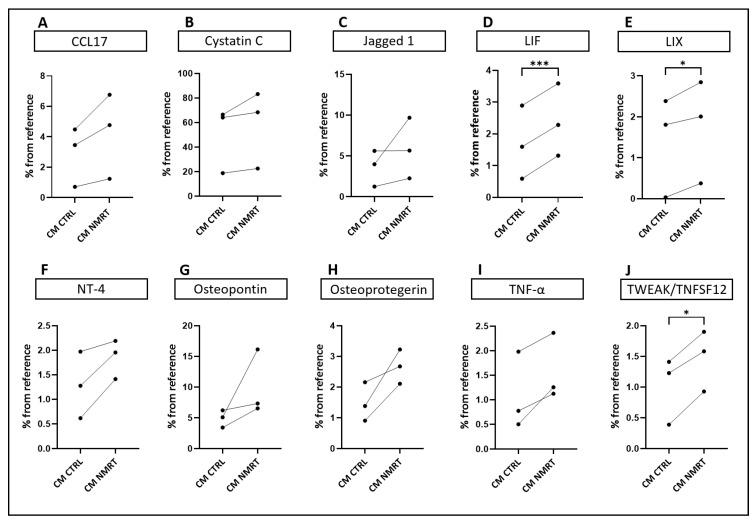
Cytokine and growth factor screening of conditioned media. (**A**) Diagrams show CCL17, (**B**) Cystatin C, (**C**) Jagged 1, (**D**) LIF, (**E**) LIX, (**F**) NT-4, (**G**) Osteopontin, (**H**) Osteoprotegerin, (**I**) TNF-α, and (**J**) TWEAK/TNFSF12 concentration measured in CM CTRL and CM NMRT; data are depicted relative to the reference signal on every membrane and as average values of measured duplicates for each donor; values that correspond to the same donor are connected by a line (*n* = 3), paired *t*-test * *p* < 0.05, *** *p* < 0.001.

Our results demonstrate the key role of ßNGF in CM NMRT for the observed enhanced neurite outgrowth in DRG neurons cultured in CM NMRT. However, a minor contribution of other factors contained in CM NMRT cannot be excluded, because DRG neurons always grew best when cultured in CM NMRT compared to the SCEM + ßNGF (see Figure 3). To verify that, we compared a panel of secreted factors in the CM NMRT, CM CTRL and pure SCEM using a proteome profiler, which can detect 79 cytokines known to support cell proliferation, growth and survival. Our results show that under NMRT or CTRL conditions, SCs produced a high number of different cytokines that were not detectable in the plain SCEM controls; a heatmap of the obtained data and two representative membranes are given under Supplementary Materials (Supplementary Figure S1A,B, Table S4). Ten cytokines turned out to be of particular interest (Figure 4 and Supplementary Table S5). CCL17, cytostatin C, jagged 1, neutrophin-4 (NT-4), osteopontin, osteoprotegerin and tumor necrosis factor α (TNF-α) showed a clear but not significant tendency in increasing expression under NMRT (Figure 4A–C,F–I). Three of the cytokines screened via the panel significantly changed their expression under NMRT. The fold change in expression (given in mean ± SD) of leukemia inhibitory factor (LIF) increased from 1.69 ± 1.15 to 2.39 ± 1.15, that for lipopolysaccharide-induced CXC chemokine (LIX) from 1.41 ± 1.22 to 1.74 ± 1.25 and for tumor necrosis factor-like weak inducer of apoptosis (TWEAK) from 1.01 ± 0.54 to 1.47 ± 0.49 (Figure 4D,E,J). The rest of the targets did not show differences but their values are given within the Supplemental Materials; whereas a detailed description of their potential role is presented in Supplementary Table S6 [36,37,38,39,40,41,42,43,44,45,46,47].

### 3.5. CM NMRT Did Not Affect the Expression or Activation of TRPV1 in Dorsal Root Ganglion Neurons

**Figure 5:**
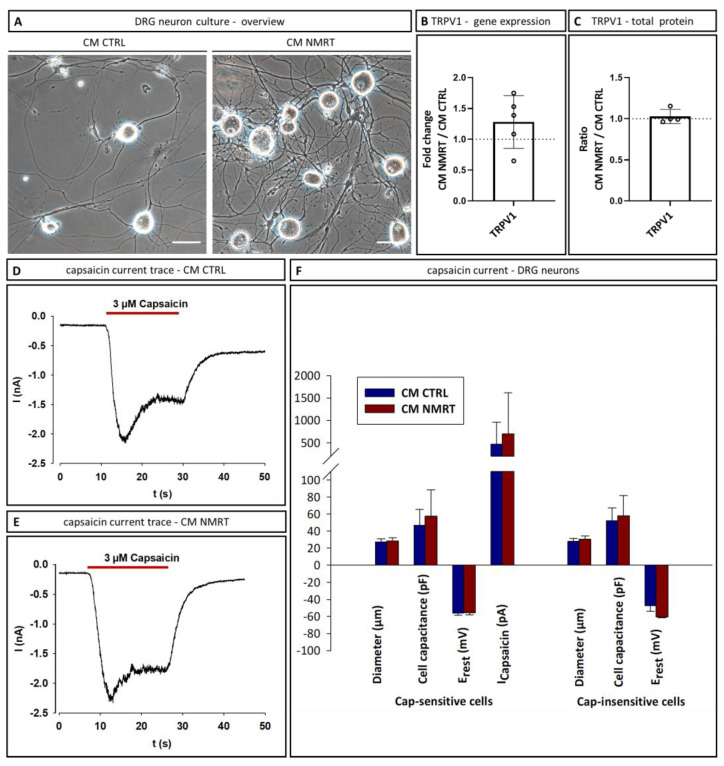
Analysis of TRPV1 expression and activation in DRG neurons cultured in CM CTRL and CM NMRT. (**A**) Representative phase contrast images illustrating the morphology of DRG neurons cultured in CM CTRL (left images) as well as CM NMRT-cultured DRG neurons (right images) after 24 h incubation. Scale bars represent 50 µm. (**B**) RT-qPCR results of TRPV1 as the gene expression fold change (*n* = 5). (**C**) Western blot analysis of TRPV1 protein isolated from whole DRG neurons cultured in CM CTRL and CM NMRT for 24 h; the diagram shows the ratio of TRPV1 between CM NMRT and CM CTRL with no differences between the two conditions; data are depicted as single values for each donor of ± SD (*n* = 4). (**D**) Representative TRPV1 current traces activated by 3 µM capsaicin at a holding potential of -80 mV in DRG neurons cultured in CM CTRL and (**E**) CM NMRT for 24–30 h. (**F**) The graph illustrates two groups of DRG neurons, capsaicin-sensitive and capsaicin-insensitive ones. No significant differences were observed in neuron diameter, cell capacitance, resting membrane potential or TRPV1 current between DRG neurons cultured in CM CTRL and CM NMRT; data are mean ± SD from 8 to 26 cells of four donors.

A crucial question for any potential future clinical use is whether the rise of growth factors and cytokines in the CM NMRT may cause an increased nociception, e.g., by affecting the expression and activation of TRPV1 [19]. Therefore, freshly isolated DRG neurons were cultured in CM CTRL and CM NMRT (Figure 5A) for 24–30 h. In a first approach, TRPV1 gene (Figure 5B) and protein (Figure 5C, Supplementary Figure S2) levels of DRG neurons were analyzed both in NMRT and CTRL conditions by RT-qPCR and western blot, and no significant differences of TRPV1 expression were found. All data can be found in Supplementary Table S7. To determine TRPV1 activation, the membrane currents were measured using the whole-cell patch clamp method. TRPV1 currents were induced by application of 3 µM capsaicin to DRG neurons cultured for 24 h in CM CTRL (Figure 5D) or CM NMRT (Figure 5E). Beside the resting membrane potential (E_rest_), also the diameter and capacitance of DRG neurons were determined. Nociceptive neurons belong to the small C-fiber or Aδ-fiber group; therefore, TRPV1 receptors are primarily expressed in small diameter fibers. However, not all small diameter neurons are nociceptive and 18 (41%) of 44 cells did not show a capsaicin-induced current. For the determination of cell diameter, only cell bodies were measured, whereas for cell capacitance, the whole surface of the neurons including neurites is essential. The diameter of DRG neurons, cell capacitance, E_rest_ and membrane current of the capsaicin sensitive and the capsaicin insensitive group did not significantly differ between CM CTRL and CM NMRT (Figure 5F). In conclusion, these results indicate that CM NMRT does not significantly alter the expression and activation of TRPV1 in DRG neurons. All data can be found in Supplementary Table S8.

### 3.6. CM NMRT Did Not Influence the Intracellular Ca^2+^ Response to Capsaicin and ATP in Dorsal Root Ganglion Neurons

**Figure 6:**
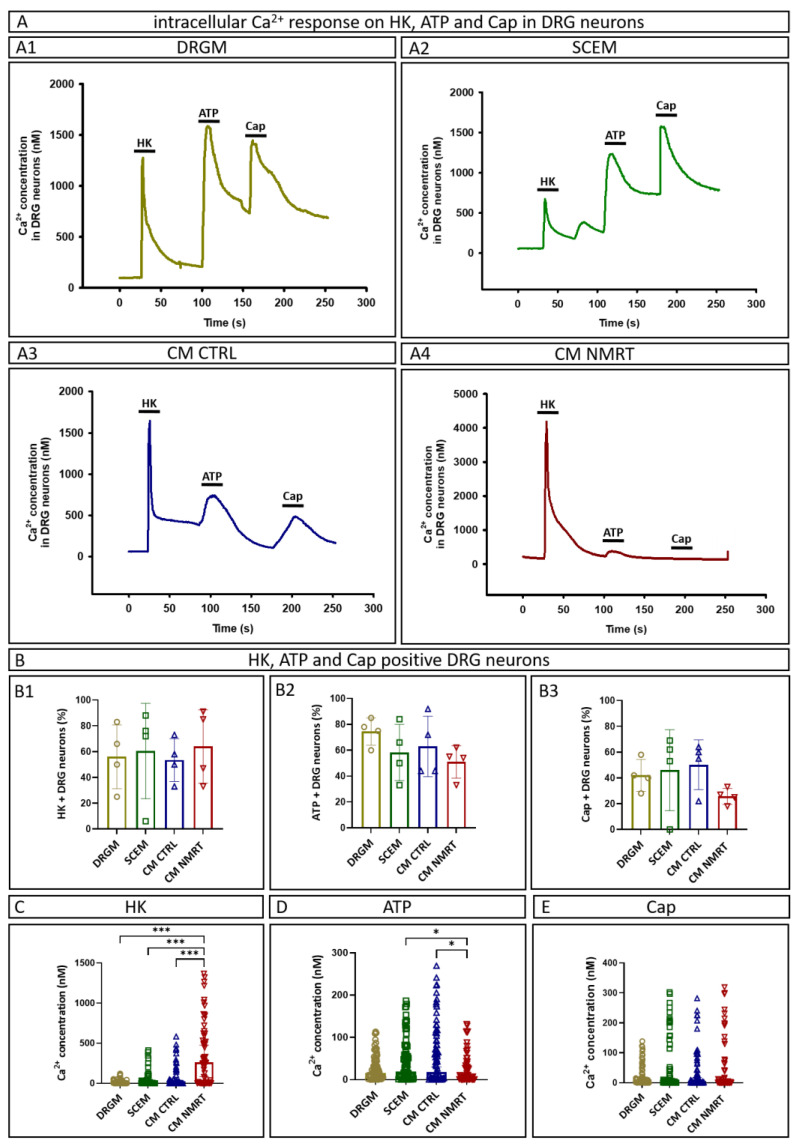
HK, ATP and Cap-induced Ca^2+^ release in DRG neurons cultivated in DRGM, SCEM, CM CTRL and CM NMRT. (**A**) Representative trace of [Ca^2+^]_i_ response to HK, ATP (10 µM) and Cap (3 µM) in a single DRG neuron cultured in (**A1**) DRGM, (**A2**) SCEM, (**A3**) CM CTRL and (**A4**) CM NMRT. (**B**) Percentage of cells reactive to HK (**B1**), ATP (**B2**) and Cap (**B3**). Analysis shows similar percentages of DRG neurons responding to HK, ATP and Cap within the four groups; data are depicted as mean ± SD (*n* = 4). (**C**) [Ca^2+^]_i_ concentration measured as a response to HK. Significantly higher [Ca^2+^]i concentration in DRG neurons cultured in CM NMRT compared to controls. (**D**) [Ca^2+^]_i_ concentration measured in response to ATP revealed a significantly higher intracellular Ca^2+^ concentration in DRG neurons cultured in SCEM and CM CTRL compared to the CM NMRT group. (**E**) [Ca^2+^]_i_ concentration measured as a response to capsaicin without significant differences between the four groups; data are depicted as single values for all donors of median + 95% CI (*n* = 4), one-way ANOVA, * *p* < 0.05, *** *p* < 0.001.

In a further approach, the influence of CM NMRT on the sensitivity of DRG neurons for activators mimicking noxious stimuli was determined, measured as increased calcium influx. DRG neurons were cultured either in DRGM, SCEM, CM CTRL or CM NMRT and [Ca^2+^]_i_ response to Cap, ATP or HK was recorded, the latter being an indicator for cell viability (Figure 6(A1–A4)). The intracellular Ca^2+^ concentration after application of one of the test substances frequently did not return to the resting value. This might be owed to the high volume-to-surface ratio of such big cells which makes it difficult for membrane pumps to remove Ca^2+^ from the cytosol. Therefore, the effect of substances was always assessed by the difference between the resting Ca^2+^ value immediately before application and the maximum value reached during application of a substance. Yet, there might be an underestimation of the absolute effect of substances that were applied later during an experiment; although due to the unchanged sequence of substance application in all the experiments, the relative changes in effects should be affected only marginally. The percentages of DRG neurons responding to HK, ATP and Cap were similar in all groups (Figure 6(B1–B3)), but [Ca^2+^]_i_ levels in response to HK were significantly higher in CM NMRT-cultured DRG neurons (Figure 6C). Interestingly, the [Ca^2+^]_i_ response to ATP was significantly higher in CM CTRL and SCEM incubated DRG neurons compared to the CM NMRT group (Figure 6D), whereas no difference between the four groups was detected in response to Cap (Figure 6E). These results demonstrate that DRG neurons cultured in CM NMRT showed the highest viability, and factors contained in CM NMRT did not alter their capsaicin sensitivity, but even reduced their sensitivity to ATP. All data can be found in Supplementary Table S9.

## 4. Discussion

There is an ongoing search for innovative treatments able to enhance peripheral nerve regeneration. Our previous in vitro study showed that the conditioned medium of NMRT-treated SCs (CM NMRT) enhanced the neurite outgrowth of DRG neurons [27], indicating a therapeutic potential of NMRT for improved regeneration of injured peripheral nerves. To provide insight into the underlying mechanisms, the current in vitro study analyzed the CM NMRT for potentially relevant mediators such as ßNGF and cytokines. Furthermore, CM NMRT-associated possible pro-nociceptive effects on cultured DRG neurons were investigated.

In our study, ßNGF levels in the supernatant of NMRT-treated SC cultures were significantly higher than without NMRT exposure. In fact, when the respective concentration of ßNGF measured in the CM NMRT (10 pg/mL) was added to unconditioned medium, it was sufficient to increase the neurite formation of DRG neurons. Vice versa, it was significantly reduced when CM NMRT was mixed with a neutralizing anti-ßNGF antibody. These results demonstrate that the previously observed effect of NMRT on DRG neuron growth was primarily mediated by the NMRT-induced increased secretion of ßNGF from SCs. Furthermore, this is in line with previous data showing that the exposure to a low-intensity magnetic field in a mouse model promotes TGF-β and associated receptor expression, which might induce NGF release [28,29,30]. Huang et al. [ ] reported that electric stimulation of SCs directly results in the release of NGF in a Ca^2+^ dependent manner. However, the frequency of the electric field used by these authors was in the range of 1–100 Hz compared to 16 kHz utilized by NMRT, so it is questionable whether these effects are comparable and share the same cellular mechanisms. Another possibility is that NMRT inhibits the release or synthesis of NO as NO is known to inhibit NGF release from various cells [48]; whether NMRT is able to modulate the NO synthesis remains to be shown.

Although the unconditioned SC medium plus added ßNGF significantly increased the outgrowth of DRG neurons, it was observed that DRG neurons still had the best growth behavior when cultured in CM NMRT. Thus, NMRT exposure of SCs may induce minor increases of various cytokines and growth factors known to be involved in neuronal survival and axonal outgrowth (for review see [49,50]). However, with the exception of cystatin C, other cytokines screened for in our assay showed relatively low expression levels, jagged 1, CCL17 and osteopontin being elevated in some samples. Cystatin C is virtually always excreted from nucleated cells, so a specific role in nerve regeneration is questionable. CCL17 is a chemokine known to attract T-cells and to be able to induce the release of NGF from mouse T cell hybridoma cell lines [36] but also from keratinocytes [51]. Whether Schwann cells can release NGF upon CCL17 stimulation remains to be shown, though oligodendrocytes of the CNS do not express the CCL17 receptor CCR4 [52]. Similarly, to CCl17, osteopontin is a chemotactic factor binding to various surface receptors and is able to activate immune response due to infections, autoimmune diseases and tissue damage [53]. Whether osteopontin is involved in myelination of peripheral nerves is not known. Jagged 1 is an activator of the notch signaling pathway and thus involved in many developmental processes and organogenesis. A beneficial effect of the injection of jagged/FC chimeric protein into injured peripheral nervous tissue has been reported and was at least partially attributed to an increase in released NGF and BDNF by SCs [54]. The release of NGF from SCs by NMRT could therefore be triggered or augmented by these factors in an autocrine or paracrine manner.

Despite their primarily pro-regenerative actions, cytokines and growth factors can also induce neurotoxic effects [55,56,57,58] and neuropathic pain [59,60]. For example, low concentrations of some cytokines protect against excitotoxic damage and are neurotrophic, whereas higher concentrations are neurotoxic [61,62]. Importantly, NGF also plays a pivotal role in modulation of nociception [17,18]. Therefore, the question arises whether the secretome of the CM NMRT may also have potential pro-nociceptive effects on DRG neurons. In our experiments, [Ca^2+^]_i_ measurements showed higher [Ca^2+^]_i_ transients due to K^+^-depolarization in CM NMRT-treated DRG cells implying that CM NMRT enhances the viability of DRG neurons, but the capsaicin-induced [Ca^2+^]_i_ response was not affected by CM NMRT treatment. These results were also confirmed by whole-cell patch clamp measurements. Currents through capsaicin-activated TRPV1 channels were not influenced by the specific culture conditions. The capacitance of the neurons, however, was slightly increased in CM NMRT-treated DRG neurons even though the measured diameters were similar in both conditions. This can be explained by the fact that the CM NMRT-treated neurons had a larger neurite network in comparison to those cultured in CM CTRL, so that even when the cell body diameter was similar, the cell surface area would be greater. In addition, the TRPV1 protein and gene expression assessed by western blot and RT-qPCR were similar between DRG neurons cultured in CM NMRT and those cultured in CM CTRL. These findings do not point to pro-nociceptive effects of NMRT in vitro.

## 5. Conclusions

In conclusion, our study identified the major mechanisms responsible for the beneficial effect of the secretome of NMRT-treated SCs on cultured DRG neuron regeneration. The effect is primarily mediated by the increased release of ßNGF and presumably supported by elevated levels of pro-regenerative cytokines. Since NMRT also increased the expression of LIF known to be regulated by TGF-β, an involvement of TGF-β signaling itself by NMRT can be considered. In addition, our results demonstrate that the supernatant of NMRT-treated SCs does not alter the sensitivity of cultured DRG neurons to substances mimicking noxious stimuli, TRPV1 receptor expression or function, and thus are encouraging to test potential pro-generative NMRT effects in future in vivo studies. Based on our present experimental results, we consider NMRT a promising treatment option that could be used to support standard therapies for peripheral nerve regeneration by the non-invasive stimulation of SCs in or near the injured nerve to promote the local production of neurotrophic factors and pro-regenerative cytokines.

## Data Availability

Available on request from corresponding author.

## Supplementary Materials

The following supporting information can be downloaded at: https://www.mdpi.com/article/10.3390/cells13181544/s1, Figure S1. Cytokine screening of conditioned media. (A) Representation of all cytokines representing % of reference spots in SCEM, CM CTRL and CM NMRT. (B) Images of the membranes of the proteome profiler incubated with SCEM (upper image), CM CTRL (middle image) and CM NMRT (lower image). Spots represent the presence of cytokines in duplicates (*n* = 3) and reference spots (arrowhead). Figure S2. Western blot membrane. Representation of TRPV1 and GAPDH protein lanes in CM CTRL and CM NMRT. Table S1: Primary and secondary antibodies. Table S2: Primer sequences used for RT-PCR. Table S3: Comparative effects of conditioned media and ßNGF presence on DRG neurons. Table S4: Summary of results—Cytokine screening, n.d.: not detected. Table S5: Increased cytokine release by NMRT-treated SC. Table S6: Potential role of cytokines found elevated in NMRT-treated SC supernatant [36,37,38,39,40,41,42,43,45,46,63]. Table S7: TRPV1 protein and gene expression in DRG neurons. Table S8: Capsaicin activation of TRPV1 channels in DRG neurons. Table S9: HK, ATP and capsaicin induced Ca^2+^ release in DRG neurons.
