# Supplementary material for: Nuclear Magnetic Resonance Treatment Induces ßNGF Release from Schwann Cells and Enhances the Neurite Growth of Dorsal Root Ganglion Neurons In Vitro
Source: Cells. 2024 Sep 13;13(18):1544. doi: 10.3390/cells13181544 (PMC11430304; doi:10.3390/cells13181544)
Supplement: Supplementary file 1 [file cells-13-01544-s001.zip › 2024 07 18 Cells NMRT paper_suppl Appendix A in template submitted.pdf]

cells

MDPI

Supplementary Figures

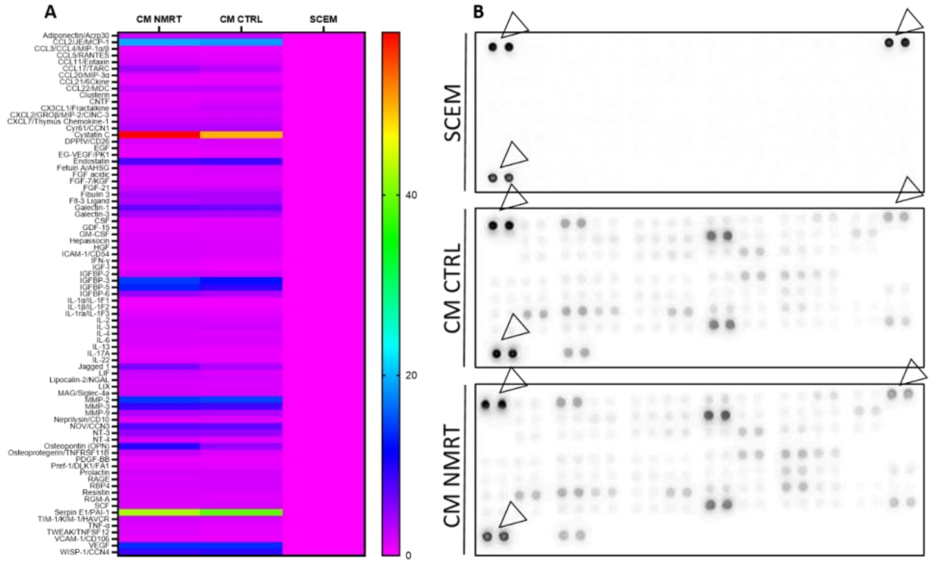

Supplementary Figure S1. Cytokine screening of conditioned media. (A) Representation of all cytokines representing % of reference spots in SCEM, CM CTRL and CM NMRT. (B) Images of the membranes of the proteome profiler incubated with SCEM (upper image), CM CTRL (middle image) and CM NMRT (lower image). Spots represent the presence of cytokines in duplicates (n=3) and reference spots (arrowhead).

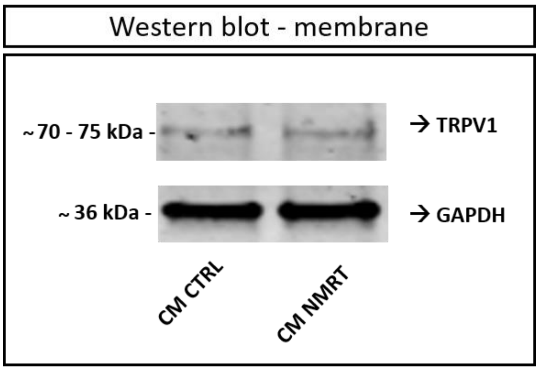

Supplementary Figure S2. Western blot membrane. Representation of TRPV1 and GAPDH protein lanes in CM CTRL and CM NMRT.

Supplementary Tables

Supplementary Table S1: Primary and secondary antibodies

Primary Antibodies				
Antigen	species	Dilution	Company	Comment
S100	rabbit	1:200	DAKO, #Z0311	Permeabilization, o.n. incubation at 4°C

Vimentin (VIME)	chicken	1:300	ThermoFisher, PA1-10003	Permeabilization, o.n. incubation at 4°C
SOX10	mouse	1:50	Santa Cruz, sc-53116	Permeabilization, o.n. incubation at 4°C
ß3Tubulin (TBB3)	mouse	1:100	Santa Cruz, B2217	Permeabilization, o.n. incubation at 4°C
NF-H	chicken	1:200	Dako, IS504	Permeabilization, o.n. incubation at 4°C
TRPV1	rabbit	1:500	Alomone labs, ACC-029	Permeabilization, o.n. incubation at 4°
GAPDH	rabbit	1:1000	Cell Signaling, #2118S	o.n. incubation at 4°
**Secondary Antibodies**				
Fluorophore	Target species	Dilution	Company	Comment
AF488	mouse	1:300	Invitrogen, A11029	1 h incubation, RT
AF594	rabbit	1:300	Invitrogen, A11012	1 h incubation, RT
DL650	chicken	1:300	Invitrogen, SA5-10073	1 h incubation, RT
LiCor	rabbit	1:15000	LiCor	1 h incubation, RT

Supplementary Table S2: Primer sequences used for RT-PCR

	**Oligonucleotide sequences**	
Target gene	forward Primer	reverse Primer
Gapdh	AGTGCCAGCCTCGTCTCATA	GATGGTGATGGGTTTCCCGT
Actb	GCAGGAGTACGATGAGTCCG	ACGCAGCTCAGTAACAGTCC
Trpv1	CTGAGAGACCTGTGCCGTTT	CACAGGCCGATAGTAGGCAG

Supplementary Table S3: Comparative effects of conditioned media and ßNGF presence on DRG neurons.

**Comparison of DRG neuron cultures and neurite features in response to conditioned media and ßNGF presence (results expressed as mean ± standard deviation)**					
	SCEM	CM CTRL	CM NMRT	SCEM + ßNGF	CM NMRT + NGF nAb
Neurons with neurites (%)	66,13 ±3,39	64,90 ± 14,71	93,82 ± 7,61	91,58 ± 7,03	70,59 ± 8,06
Neurite number / DRG neuron	1,02 ± 0,14	0,97 ± 0,27	2,03 ± 0,39	1,68 ± 0,25	1,10 ± 0,11
Neurite length (µm)	91,65 ± 9,23	111,41 ± 18,33	214,37 ± 35,67	158,04 ± 21,51	102,97 ± 10,00
Number of branching points / DRG neuron	0,43 ± 0,05	0,46 ± 0,23	1,65 ± 0,49	1,17 ± 0,49	0,42 ± 0,12
TBB3 (AFI) / DRG neuron	0,24 ± 0,14	0,20 ± 0,06	0,53 ± 0,31	0,33 ± 0,14	0,25 ± 0,11
NF-H (AFI) / DRG neuron	0,27 ± 0,13	0,24 ± 0,07	0,59 ± 0,33	0,36 ± 0,18	0,30 ± 0,18

Supplementary Table S4: Summary of results – Cytokine screening, n.d.: not detected

	Cytokine screening (% of reference spots, results expressed as means ± standard deviation)		
Cytokine	SCEM	CM CTRL	CM NMRT
Adiponectin/Acrp30	n.d.	2,43 ± 0,57	2,36 ± 0,32
CCL2/JE/MCP-1	n.d	18,84 ± 4,86	19,42 ± 7,50
CCL3/CCL4/MIP-1α/β	n.d	0,82 ± 0,40	0,83 ± 0,24
CCL5/RANTES	n.d	1,11 ± 0,62	1,21 ± 0,30
CCL11/Eotaxin	n.d	1,49 ± 0,56	1,79 ± 0,14
CCL17/TARC	n.d	2,88 ± 1,95	4,26 ± 2,80
CCL20/MIP-3α	n.d	1,02 ± 0,51	1,25 ± 0,41
CCL21/6Ckine	n.d	0,88 ± 0,43	0,80 ± 0,36
CCL22/MDC	n.d	2,77 ±1,34	2,86 ± 1,75
Clusterin	n.d	1,19 ± 0,25	1,20 ± 0,36
CNTF	n.d	1,73 ± 0,43	1,27 ± 0,18
CX3CL1/Fractalkine	n.d	2,33 ± 0,52	1,95 ± 0,57
CXCL2/GROβ/MIP-2/CINC-3	n.d	1,82 ± 1,61	1,64 ± 1,56
CXCL7/Thymus Chemokine-1	n.d	2,46 ± 0,93	2,27 ± 0,23
Cyr61/CCN1	n.d	3,29 ± 0,98	3,24 ± 0,41
Cystatin C	n.d	49,80 ± 26,86	58,03 ± 31,64
DPPIV/CD26	n.d	1,73 ± 0,85	1,64 ± 0,43
EGF	n.d	1,28 ± 0,57	0,99 ± 0,49
EG-VEGF/PK1	n.d	0,94 ± 0,26	0,85 ± 0,55
Endostatin	n.d	8,70 ± 8,52	7,97 ± 7,41
Fetuin A/AHSG	n.d	1,86 ± 0,77	1,66 ± 0,69
FGF acidic	n.d	1,51 ± 0,77	1,43 ± 0,49
FGF-7/KGF	n.d	1,27 ± 0,67	1,22 ± 0,22
FGF-21	n.d	1,94 ± 1,13	1,98 ± 0,58
Fibulin 3	n.d	3,56 ± 0,99	3,64 ± 0,41
Flt-3 Ligand	n.d	3,67 ± 2,30	3,14 ± 1,72
Galectin-1	n.d	6,55 ± 4,14	6,83 ± 4,08
Galectin-3	n.d	3,57 ± 1,18	3,73 ± 1,31
CSF	n.d	1,19 ± 0,52	1,27 ± 0,56
GDF-15	n.d	1,28 ± 0,87	1,13 ± 0,60
GM-CSF	n.d	1,37 ± 0,42	1,52 ± 0,09
Hepassocin	n.d	1,56 ± 0,40	1,72 ± 0,18
HGF	n.d	1,55 ± 1,27	1,78 ± 1,24
ICAM-1/CD54	n.d	1,52 ± 0,58	1,72 ± 0,23
IFN-γ	n.d	1,18 ± 0,40	1,40 ± 0,28
IGF-I	n.d	0,63 ± 0,30	0,90 ± 0,07

IGFBP-2	n.d	1,83 ± 0,90	2,01 ± 0,57
IGFBP-3	n.d	12,21 ± 4,35	14,14 ± 9,05
IGFBP-5	n.d	9,39 ± 5,12	13,08 ± 7,48
IGFBP-6	n.d	3,52 ± 1,00	4,56 ± 2,45
IL-1α/IL-1F1	n.d	0,55 ± 0,70	0,62 ± 0,71
IL-1β/IL-1F2	n.d	0,55 ± 0,67	0,66 ± 0,82
IL-1ra/IL-1F3	n.d	1,21 ± 0,54	1,32 ± 0,24
IL-2	n.d	1,70 ± 0,66	1,80 ± 0,23
IL-3	n.d	2,07 ± 1,07	1,85 ± 0,29
IL-4	n.d	1,49 ± 0,67	1,49 ± 0,12
IL-6	n.d	1,64 ± 1,03	1,77 ± 0,50
IL-13	n.d	1,19 ± 0,64	1,35 ± 0,19
IL-17A	n.d	0,90 ± 0,84	0,92 ± 0,60
IL-22	n.d	1,07 ± 0,53	1,24 ± 0,31
Jagged 1	n.d	3,61 ± 2,21	5,86 ± 3,72
LIF	n.d	1,69 ± 1,15	2,40 ± 1,14
Lipocalin-2/NGAL	n.d	1,88 ± 2,70	1,88 ± 2,68
LIX	n.d	1,41 ± 1,23	1,74 ± 1,25
MAG/Siglec-4a	n.d	1,81 ± 0,65	1,93 ± 0,32
MMP-2	n.d	14,49 ± 1,61	14,08 ± 2,24
MMP-3	n.d	8,60 ± 10,41	9,03 ± 10,75
MMP-9	n.d	3,35 ± 4,15	3,52 ± 3,78
Neprilysin/CD10	n.d	1,28 ± 0,67	1,37 ± 0,13
NOV/CCN3	n.d	7,11 ± 4,85	7,30 ± 4,49
NT-3	n.d	4,71 ± 2,64	4,74 ± 2,45
NT-4	n.d	1,29 ± 0,68	1,85 ± 0,40
Osteopontin (OPN)	n.d	4,90 ± 1,41	10,00 ± 5,34
Osteoprotegerin/TNFRSF11B	n.d	1,48 ± 0,63	2,67 ± 0,56
PDGF-BB	n.d	0,88 ± 0,76	0,96 ± 0,63
Pref-1/DLK1/FA1	n.d	1,14 ± 0,86	1,35 ± 0,77
Prolactin	n.d	1,95 ± 1,05	2,02 ± 0,41
RAGE	n.d	1,75 ± 0,80	1,64 ± 0,10
RBP4	n.d	1,97 ± 1,85	1,92 ± 1,21
Resistin	n.d	1,61 ± 0,82	1,64 ± 0,14
RGM-A	n.d	1,41 ± 0,83	1,52 ± 0,29
SCF	n.d	1,67 ± 1,20	1,62 ± 0,49
Serpin E1/PAI-1	n.d	39,03 ± 13,20	42,30 ± 18,34
TIM-1/KIM-1/HAVCR	n.d	1,69 ± 0,75	2,01 ± 0,32
TNF-α	n.d	1,08 ± 0,79	1,58 ± 0,68
TWEAK/TNFSF12	n.d	1,01 ± 0,55	1,47 ± 0,50
VCAM-1/CD106	n.d	0,78 ± 0,44	0,87 ± 0,09

VEGF	n.d	13,48 ± 11,00	13,67 ± 8,06
WISP-1/CCN4	n.d	9,51 ± 9,84	8,83 ± 7,83

Supplementary Table S5: Increased cytokine release by NMRT-treated SC

	Cytokine screening (% von reference spots)					
	CM CTRL			CM NMRT		
Cytokine	#1	#2	#3	#1	#2	#3
CCL17/TARC	4,48	3,45	0,71	6,76	4,77	1,23
Cystatin C	66,49	64,09	18,81	83,21	68,36	22,52
Jagged 1	3,98	1,24	5,62	9,68	2,25	5,66
LIF	1,59	0,59	2,89	2,28	1,32	3,59
LIX	2,38	0,03	1,81	2,84	0,38	2,01
NT-4	1,97	0,62	1,28	2,19	1,41	1,96
Osteopontin (OPN)	5,08	3,40	6,21	16,15	6,52	7,33
Osteoprotegerin/TNFRSF11B	1,38	0,91	2,16	3,22	2,11	2,67
TNF-α	1,98	0,50	0,77	2,36	1,26	1,12
TWEAK/TNFSF12	1,41	0,39	1,23	1,90	0,93	1,58

Supplementary Table S6: Potential role of cytokines found elevated in NMRT-treated SC supernatant

Cytokine	Potential role
CCL17/TARC (C-C Motif Chemokine Ligand 17)	accelerates wound healing, mainly by enhancing fibroblast migration, and enhanced nerve growth factor (NGF) production [23]
Cystatin C	neuroprotection, regulates cell proliferation and promotion of the mitogenic activity of cells [24]
Jagged 1	cell survival, inhibiting apoptosis, and driving cell proliferation [25]
LIF (Leukemia Inhibitory Factor)	neurotrophic properties by promoting neuronal survival and axon growth [26-28]
LIX, CXCL5 (Lipopolysaccharide-induced CXC Chemokine)	promotes cell survival and axonal regeneration [29]
NT-4 (Neurotrophin-4)	growth factor with key roles in the survival, development, and function of neurons [30]
Osteoprotegerin (OPG, osteoclastogenesis inhibitory factor (OCIF))	facilitating recovery of the organism after injury and encourage cell migration [31]
Osteopontin (OPN)	regulation of bone remodeling and neurite outgrowth [32]
TNF-α (tumor necrosis factor α)	besides its regulatory role on immune cells TNF-α has positive effects on myelin-forming cells by inducing gene transactivation, stimulates the production of NGF [33]
TWEAK/TNFSF12 (tumor necrosis factor-like weak inducer of apoptosis)	multifunctional cytokine suggested to play a beneficial role for tissue repair after acute injury [34]

Supplementary Table S7: TRPV1 protein and gene expression in DRG neurons

TRPV1 protein and gene expression in DRG neurons		
	TRPV1 total protein (ratio CM NMRT / CM CTRL)	TRPV1 gene expression (fold change)
CM NMRT/CM CTRL	1,02±0,08	1,28±0,42

Supplementary Table S8: Capsaicin activation of TRPV1 channels in DRG neurons

Capsaicin activation of TRPV1 channels in DRG neurons (results expressed as mean + standard deviation)				
	Diameter (μM)	Cell capacitance (pF)	E_{rest} (mV)	I_{Cap} (pA)
CM CTRL	27,5±3,73	48,79±17,57	-53,38±12,60	462,11±496,07
CM NMRT	29,5±4,05	58,69±29,07	-56,63±10,47	749,07±946,95

Supplementary Table S9: HK, ATP and capsaicin induced Ca^{2+} release in DRG neurons

HK, ATP and capsaicin induced Ca^{2+} release in DRG neurons (results expressed as mean + standard deviation)				
	DRGM	SCEM	CM CTRL	CM NMRT
HK+ DRG neurons (%)	56±24,67	60,5±36,96	53,5±16,66	64±28,40
ATP+ DRG neurons (%)	74,5±10,53	58,25±21,82	63±23,40	51±12,51
Cap+ DRG neurons (%)	42±12,33	46±31,36	50,25±19,19	25,75±6,18
[Ca2]i response to HK (nM)	127,45±232,37	302,86±548,97	349,16±574,53	671,31±895,99
[Ca2]i response to ATP (nM)	69,40±174,88	79,63±113,90	203,29±397,21	60,95±95,65
[Ca2]i response to Cap (nM)	109,29±205,36	171,58±343,20	180,66±351,04	151,08±302,00
